# Recruitment and Retention of Healthy Women with Obesity for a Psychophysiological Study before and After Weight Loss: Insights, Challenges, and Suggestions

**Published:** 2021-02-28

**Authors:** Dharini M. Bhammar, Vipa Bernhardt, Jonathon L. Stickford, Charles Miller, Tony G. Babb

**Affiliations:** 1 Department of Kinesiology and Nutrition Sciences, School of Integrated Health Sciences, University of Nevada, USA; 2 Department of Health and Human Performance, Texas A&M University-Commerce, USA; 3 Exercise and Respiratory Physiology Laboratory, Department of Health and Exercise Science, Appalachian State University, USA; 4 Department of CV Surgery, University of Texas Health Sciences Center, USA; 5 Institute for Exercise and Environmental Medicine, Texas Health Presbyterian Hospital Dallas and UT Southwestern Medical Center, USA

## Abstract

**Objective:**

The objective of this paper is to present data on participant recruitment, retention, and weight loss success during a psychophysiological study in women with obesity.

**Methods:**

Volunteers were women with obesity, 20 – 45 yr, with a BMI between 30 – 45 kg/m^2^. The study was approximately 20 weeks in duration, including a 12-week weight loss program.

**Results:**

Recruitment was not completed until 8 months past the original projected date of 12 months. The study was not completed until 11 months past the original projected completion date of 14 months. On average 4.4 ± 2.1 (mean ± SD) volunteers were consented per month (N = 99) and 2.5 ± 1.1 participants started the weight loss program per month. 24% of consented volunteers were lost due to exclusion criteria, withdrawals, and unresponsive behavior before starting the weight loss program. Attrition of participants who started the weight loss program was 45%. Only 11% of those who started the program were unable to lose weight (N = 6).

**Conclusion:**

Recruiting and/or weight loss success do not always present the most challenging aspects of completing a psychophysiological weight loss intervention. While participant attrition during a weight loss program can occur for a wide range of reasons supportive efforts in the early phases of the intervention may maximize retention.

## Introduction

Attrition rates in weight loss studies are notoriously high and success rates in weight loss studies are extremely low. However, interventional trials are an important part of many human research studies [1,2]. Two of the major challenges in successfully completing interventional trials are participant recruitment and retention [1,3,4]. Participant recruitment and retention may be influenced by the unique characteristics of the participant recruited, including age, race, socioeconomic status, employment status, smoking status, and overall health [5]. In addition, practical barriers, or logistics such as time and family commitments as well as lower initial weight loss can affect retention in studies [6]. Participant retention could be improved through financial incentives, multicomponent strategies such as the inclusion of home environment changes or dietician-assistant behavior change plan, and self-monitoring technology with feedback [7]. We present our experiences in attempting to recruit and retain otherwise healthy, women with obesity for a psychophysiological study before and after successful weight loss. The overall experimental objective of the study was to investigate whether weight loss can ameliorate exertional dyspnea in otherwise healthy women with obesity [8,9]. However, the purpose for this article is not to present the results of that study. Although our study was unique in the fact that the participants had to lose weight in order to assess the effectiveness of weight loss as a treatment for exertional dyspnea, the aim of this paper is to describe our recruitment process, retention challenges, weight loss success, and strategies to recruitment, retention, and weight loss procedures over the course of the trial. Our intent is to identify the difficulties in running a physiological interventional trial to assess the psycho-behavioral perception of breathlessness and to assist others who are planning similar types of weight loss studies in adults with obesity.

## Methods

### Participants

We screened female volunteers with obesity between the ages of 20-45 years over the phone and during their first visit to the laboratory where they completed a medical history form. We included participants with a BMI between 30-45 kg/m^2^, normal pulmonary function, no history of smoking, no history or evidence of heart disease, no history of uncontrolled hypertension, no history of significant mental illness, no significant substance or alcohol abuse, no metabolic disorders, no history of sleep disordered breathing, no musculoskeletal abnormalities that would preclude exercise, and no current medications that could interfere with exercise capacity. Participants participating in regular vigorous exercise (i.e., exercise more than 2x/wk) during the last 6 months were also excluded. No exclusion was made on the basis of race/ethnic background or socioeconomic status. Written informed consent was obtained before participation. (UT Southwestern IRB approval #STU122010-108). The participants had to be available for testing during regular work hours and had to be able to meet three times a week with a personal trainer at our Cardiovascular Fitness Center for 12 weeks. Appointment times for meeting with the trainer were individually arranged for the participant’s convenience as long as it was during the center’s operating hours (typically Monday through Saturday between 5 am and 8 pm). The laboratory and fitness center are located approximately 6 miles northeast of downtown Dallas, TX in an easily accessible part of the city with plenty of available free parking.

### Experimental design

The present study was designed as a pre-post interventional experiment. Volunteers were expected to commit to participating in the study for approximately 20 weeks, including comprehensive testing before and after successful weight loss. The women with obesity were assigned to one of two groups according to their rating of perceived breathlessness (RPB, 0 – 10 Borg scale) during minute 6 of a submaximal constant load exercise test at 60 W (see below). Those with an RPB ≤ 2 were designated as women with obesity with no or mild dyspnea on exertion (−DOE) and those with an RPB ≥ 4 were designated as women with obesity with strong dyspnea on exertion (+DOE). Those women with an RPB = 3 were excluded from the study in order to better delineate differences between the +DOE and −DOE groups. The grouping was based on our previous findings and studies [10]. Based on previous findings [11], we expected approximately one-third of participants consented to be excluded due to an RPB of 3. The overall experimental objective of the study was to investigate whether weight loss can ameliorate exertional dyspnea in otherwise healthy women with obesity and whether the change in exertional dyspnea was related to weight loss-induced changes in body composition, pulmonary function, and/or cardiopulmonary responses to exercise including measurements of breathing mechanics or the work of breathing [8,9].

### Testing procedures

Testing took place during four separate visits. Visit 1 included measurement of height, weight, body circumferences (neck, chest, waist, and hips), and determination of body composition by underwater weighing, and pulmonary function testing (including spirometer, maximal voluntary ventilation, lung volumes, diffusing capacity, and maximal inspiratory and expiratory pressures). During visit 2 participants performed submaximal constant load cycling exercise at 60 W and an incremental maximal cardiopulmonary exercise test. Visit 3 included the determination of the oxygen cost of breathing by eucapnic voluntary hyperpnoea. During visit 4, magnetic resonance images were taken for chest and abdominal fat distribution measurements. Visits lasted between 1 – 3.5 hr. and it usual took 4 weeks to complete all four pre-weight loss visits (i.e. about 1 visit per week). Participants underwent the same testing procedures after the 12-week weight loss program. These procedures have been described in detail previously [8,9]. Participants were compensated $50/visit for visits 1 and 4, $60 for visit 2, and $100 for visit 3; the total compensation for completing the study was $520. Although no separate compensation was provided for participation in the weight loss program, the meal plan, dietician consult, and the supervised resistance training program were provided at no cost to the participants.

### Weight loss program

Biometrics^©^ Nutrition & Fitness (Rockville, MD, USA) is a weight loss program combining dietary restriction and resistance exercise. A low-calorie, nutritionally balanced meal plan was customized for each participant based on individual food preferences and consisted of three larger meals (breakfast, lunch, dinner) and three snacks between meals with the goal of achieving a weight loss of 1 – 2 lb/week. After the initial calorie intake goal was set based on the participants age, height, and weight, the calorie intake was reduced by 100 kcal every 2 weeks, but never allowed to fall below 1000 kcal. The macronutrient composition of the diet was approximately 60% carbohydrates, 20% protein, and 20% fat. Participants were provided with weekly shopping lists for all meals and detailed information on food measurements and caloric counts. Participants were required to prepare all meals themselves with only few allowed substitutions. Participants met with a registered dietician for a consultation before beginning the meal plan. The resistance exercise was performed three times per week with a personal trainer and consisted of slow-movement (i.e., 10 sec concentric phase) resistive exercises to minimize the loss of lean body mass and thus increase basal metabolic rate. Participants found the exercises to be ‘difficult’, especially if they had not lifted weights in the past. All participants met with the lead personal trainer to discuss the program prior to initiating the weight loss program and to have preliminary measurements of body circumferences, including arm and leg circumferences. Most participants also met with a registered dietician for guidance on implementing the weight loss program. All workouts took place at the Texas Health Finley Ewing Cardiovascular and Fitness Center Dallas, which is located in the building adjacent to our laboratories. Progress logs maintained by the personal trainers were reviewed weekly by research staff.

### Recruitment

Volunteers were recruited from the Dallas, Texas metroplex (i.e., approximately 4 million people) by advertisement flyers, search through our internal participant database, emails to employees of Texas Health Resources, placement of study advertisements on the Texas Health Presbyterian Hospital Dallas website for research/clinical trials, study listings on the UT Southwestern website for clinical trials, e-sign monitor displays of studies at Texas Health Presbyterian Hospital Dallas and the Finley Ewing Cardiovascular and Fitness Center, advertisements in The Presbyterian “*Scoop*” an internal hospital newsletter, advertisements on *Craigslist*, advertisements on *Dallas Back page*, emails through *Sunshine Day* a registry of people who desire to participate in research trials, paid advertisements in the *Dallas Observer*, paid advertisements on *Center Watch*, and word-of-mouth. No overall recruitment plan was implemented other than free options were utilized first, which we had used in previous studies of obesity, and new options were added when responses to current advertisements decreased and more participants were needed. Participants enrolled on a first-come first-served basis. We originally set our recruitment goal at two participants completing pre-testing per month with both participants finishing the weight loss program three months later (Table 1). Although we had some preliminary data collected in advance, full recruitment began in December 2010. We calculated that if three volunteers were consented each month and assuming one out of every three would be excluded because their RPB rating was 3, recruitment of 24 participants would be completed by November 2011 (12 months). We calculated that if two participants would enter the 12 week weight loss program the month they were consented, we would have 12 participants per group completed by February 2012 (15 months total) at which point we would be done with data collection (N = 24).

### Statistical analysis

Differences in characteristics between participants who completed, withdrew, or were disqualified from the study were assessed with a one-way analysis of variance. The Chi square test was used to examine proportional differences by race and ethnicity. *P*<0.05 was considered statistically significant.

## Results

**Figure 1:**
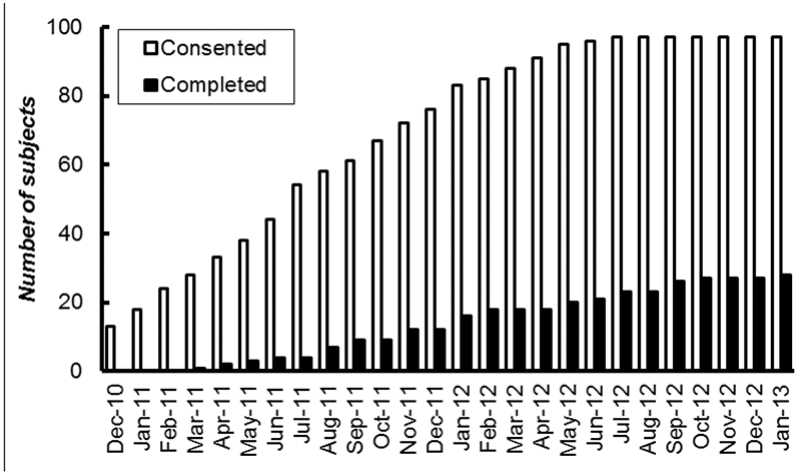
Cumulative number of participants who were consented and who completed the weight loss program.

**Figure 2:**
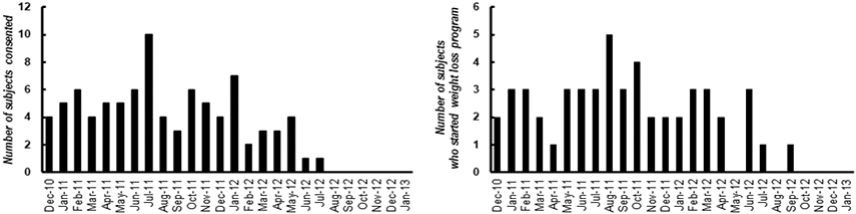
A) Number of participants consented each month. B) Number of participants who started the weight loss program each month.

**Figure 3:**
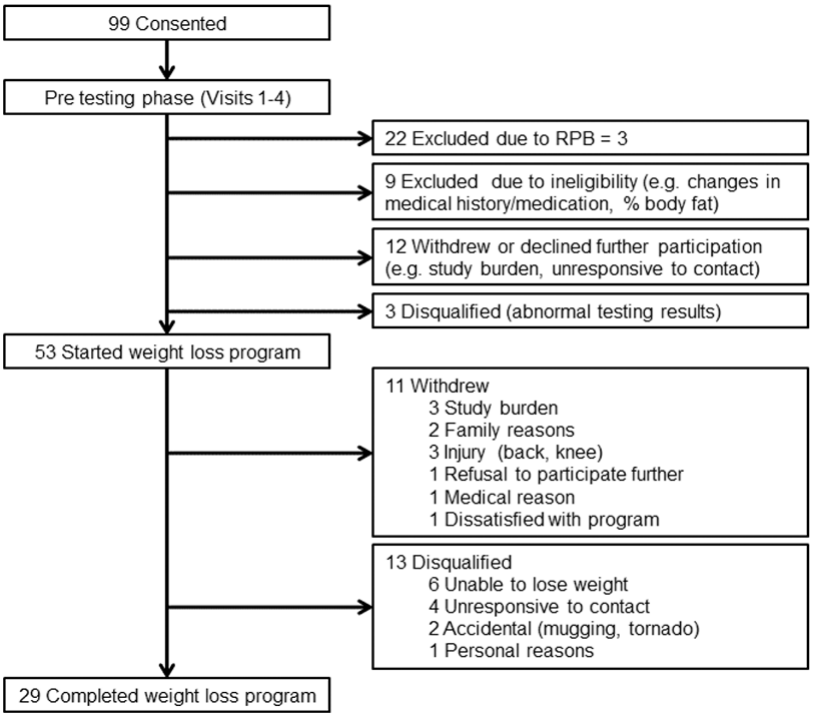
Study enrollment and retention of participants through the weight loss intervention. Reasons for exclusion, withdrawal, and disqualification are listed on the right.

Recruitment was not completed until July 2012 or 8 months past our original projected date (20 months total). The study was not completed until January 2013 or 11 months past our original projected completion date (25 months total) (Figure 1). However, the delay in completing the study was not from low recruitment numbers. From December 2010 through July 2012, we consented an average of 4.4 ± 2.1 (mean ± SD) participants per month (Figure 2A). This was higher than the three recruits per month that we had used for our projections. Furthermore, the delay in completing the study was not due to entering too few participants into the weight loss program each month. On average, 2.5 ± 1.1 participants started the weight loss program per month, which is slightly greater than the 2 participants per month we originally projected (Figure 2B). We expected to exclude one-third of consented volunteers due to an RPB rating of 3 but it was only 22% (N = 22) (Figure 3). We lost another 24% of consented participants due to exclusion criteria, withdrawals, and unresponsive behavior before starting the weight loss program. We did not expect this loss of participants to be so large. We had anticipated the loss to be closer to 10% of consented participants. We were able to compensate for the loss of volunteers before weight loss by increasing the number of participants we recruited and the length of the study. Attrition of participants after starting weight loss meant we had to continue to recruit participants much longer than expected. By our expected completion date of February 2012, we had only 18 participants completed. Also, we did not want to stop recruitment when the 24^th^ participant entered weight loss because we were not sure those participants would actually complete the weight loss program. By the time we had 24 completed, 5 participants were currently in testing and/or in the weight loss phase of the study, and it took another four months to finish and complete post-weight loss testing on these participants. The various reasons for dropping out of the weight loss program are listed in Figure 3. We had anticipated that 20% of participants enrolled in the weight loss program would be unsuccessful at losing weight. In fact, only 11% of the 53 who started weight loss were unable to lose weight in the first 6 weeks of the program (N = 6). However, there were many other reasons for dropping out of the weight loss program. Some of these problems were not anticipated at all (e.g., family members dissatisfied with meals or with the spouse being away from the home too much).

**Figure 4:**
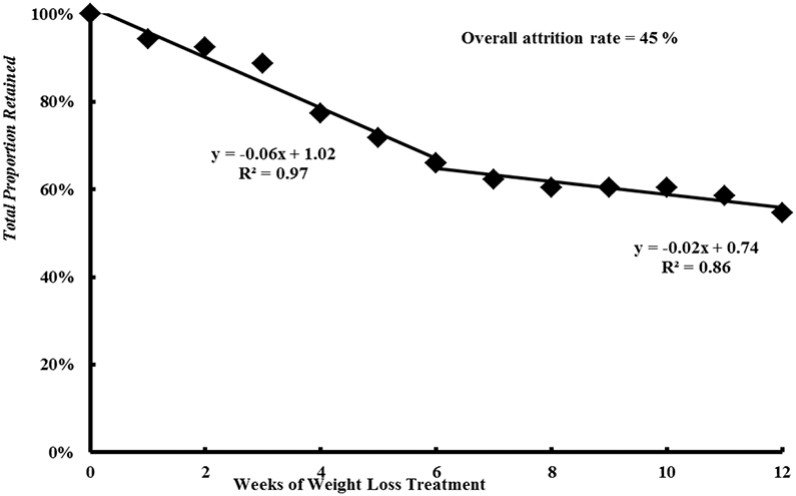
Retention and attrition rate through the weight loss intervention.

**Figure 5:**
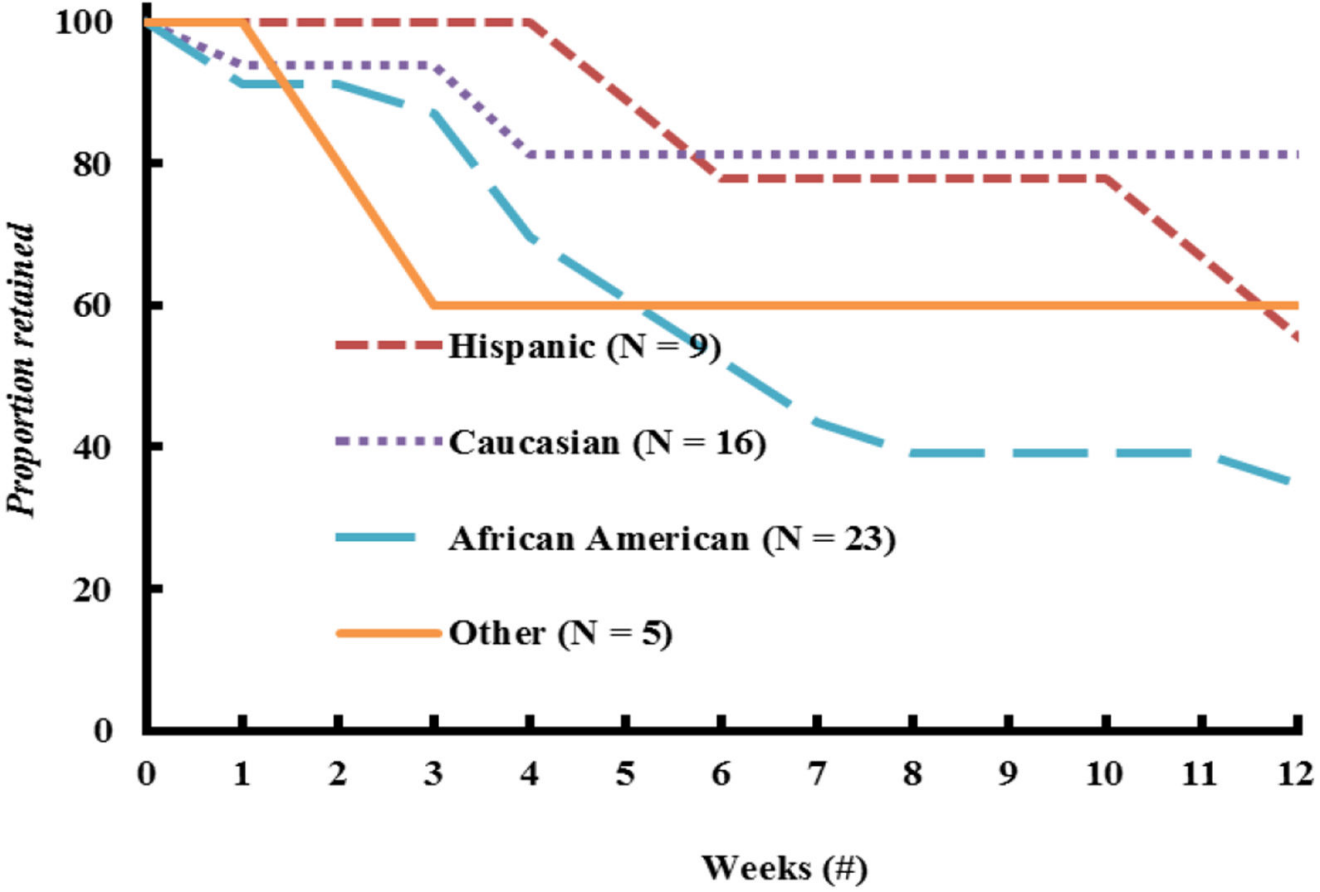
Retention and attrition rate through the weight loss intervention by race and ethnicity. Numbers in parentheses denote participants who started the weight loss program. The “other” category includes Asian (N = 3), American Indian (N = 1), and 2 or more races (N = 1).

Characteristics of participants who completed, withdrew, or were disqualified from the study are presented in Table 2. There were no differences in age, anthropometrics, body composition, or fat distribution between participants who completed, withdrew, or was disqualified from the study. Forty eight percent of African American participants were disqualified, which was significantly different from the proportion of African American participants who either completed or withdrew from the trial (*P*<0.05). Overall, we lost 45% of the participants who entered the weight loss program (Figure 4). This rate was much larger than observed for fitness center clients who pay for the program and much greater than we anticipated. The total proportion of participants retained is shown in Figure 4. These data suggest that attrition rate was much less over the second half (i.e., 6 – 12 weeks) than in the first half (0 – 6 weeks) of the intervention. This makes practical sense since those who were either successful with weight loss or found the program tolerable over the first 6 weeks were likely to stay in the program until the end. Figure 5 shows that the attrition rate appeared to be higher in African American participants at 65%. It should be noted that African American participants were 43% of our total participant population.

## Discussion

The major finding of this study is that it is not necessarily recruiting or weight loss success that delays study completion or complicates a weight loss study in otherwise healthy obese women. We found that retention during the weight loss program was the primary reason for delay in completing the study, but the reasons for attrition were not as usually expected (i.e., weight loss success). Besides meeting recruitment projections, investigators must set reasonable study schedules and accurately anticipate the impact of exclusion rates, withdrawals, study burden demands, conflicts with participant employment, facility locations, family interactions, and related medical issues, which all can affect study attrition rates [1,3,12]. Overall, recruitment was not a limiting factor in the study, although we did have to consent many more participants than we originally anticipated. The expenses related to participant compensation and extended staff salary support were increased due to these problems. Detailed attention to developing an *a priori* recruitment plan including both passive and active recruiting might have helped us with the initial recruitment efforts [1]. A better understanding of the number of participants we would lose before beginning the weight loss program would have been helpful in planning the timing of the study and in estimating the budget. Another problem we identified in the beginning of the study was that it took a month or more to complete pre-testing of a participant before we could get them entered into the weight loss program. We had estimated that each participant would have at least one test per week and would enter the weight loss program within one month of consenting. This turned out not to be reasonable since not every participant could complete one test per week due to other commitments; thus pre-testing could take more than a month to complete. We also needed to get a meal plan ordered and delivered to the participant before meeting with the trainer. Furthermore, it took time to schedule meeting times with the dietician and the individual trainers, and to schedule appointments for the magnetic resonance imaging (i.e., body composition measurements), which we had not appropriately projected. Moreover, we extended the weight loss program for some participants (N = 3) to give them more time to obtain the weight loss goal, which also delayed the completion of the study. Since the goal of our study was not to test the intent-to-treat obesity but to investigate the effects of weight loss on breathlessness during exercise, we could only test participants who lost weight. When planning the timing of studies similar to ours, it is imperative to assess potential delays at each step of the study. In our case delays occurred due to testing time (i.e., pre and post weight loss), on-boarding time (i.e., ordering meal plan, arranging other consultations), and time to actually lose weight. While we expected to exclude one-third of participants recruited (i.e., those with an RPB rating of 3), we did not expect to lose so many participants to exclusion criteria, withdrawals, or loss of interest in the study before starting the weight loss program. However, for the most part, these exclusions were outside of our control. We estimated that 10% of participants might be excluded due to abnormal pulmonary function tests (e.g., asthma) or abnormal exercise tests (e.g., exercise induced hypertension), which is a plan estimate similar to that used by others [1]. However, some participants simply lost interest in the study before we could get them into the weight loss program and/or they realized the time demand was too much even during the 4 – 6 weeks of pre-testing. It was also not unusual for participants to have a change in job responsibilities (e.g., change in hours or work location), family member issues (e.g., major illness in the family), a cold or flu that would prolong testing, and/or loss of interest about study participation. Overall, we lost 24% of consented participants during the run-in period of pre-testing due to these issues. In response to these matters, we queried subsequent participants more about the stability of their jobs (e.g., possibility of transfer, extended or changed hours, promotion, etc.), commitment to a demanding study, upcoming travel arrangements, and their home responsibilities. We also asked if it was reasonable for them to undertake the responsibilities of being in a 20-week study that required them to come to the fitness facility 3 days per week. In fact, it may be up to the investigators to help some volunteers understand that the study may not be reasonable for them. This point is reiterated by Türk et al. [13] where 37.5% of screened obese asthmatic patients were not included in the study due to “no time” or “logistic problems”. It is unclear if these alterations to our consenting practices actually helped to make participants more aware of the time they were committing to and decreased our losses. Similar problems also contributed to attrition during the weight loss program. We did not anticipate that it would be these issues rather than the failure to lose weight that would account for so many participants dropping out. We thought adult women with obesity would be excited to participate in a weight loss program free-of-charge and that we would have little problem other than weight loss issues. However, the reasons for withdrawal were quite wide ranging. Since the age requirements for the study were between 20 and 45 years, many of our participants were working mothers, scheduling visits and workouts were challenging at times for them. Changes at work were also a large problem for them. This study took place during our nation’s recent financial crisis. With high unemployment, we could certainly understand how concerns with work were very serious for many of our participants. While we did compensate participants for their pre-testing and post-testing visits, we did not provide additional compensation during the weight loss program. We elected not to provide additional compensation during the 12-week program because the program itself was expensive (i.e., cost of the Biometrics meal plan, dietician consult, and personal trainer for resistance training) and offered to participants at no cost. However, providing some additional incentive may be something that future studies could consider as a strategy for increased retention. Previous studies have provided large incentives for attending milestone sessions or small weekly incentives for participants adhere to the program and meet the weight loss goals, both of which have improved retention [14-16]. Another factor that could be considered is the provision of child care during the training sessions, which we were unable to provide. Honas et al. reported that African Americans had a 68% increased risk of drop out compared with Caucasians during a 16 week clinic-based weight loss program. African Americans made up 6% of the sample in Honas et al. Other predictors for attrition were divorced status compared with married status and younger age (i.e.,<50 yr compared with 51 – 60 yr) [17]. We also observed increased attrition among African American participants, although African Americans were a much larger proportion (i.e., 43%) of the sample that began our weight loss program. Chang et al. examined predictors in African American (52%) and Caucasian (48%) participants and did not find race as a significant predictor for attrition in their theory-based culturally sensitive 10 week weight loss intervention [18]. Tailoring the intervention for cultural sensitivity could be a factor to consider in future research to minimize differential attrition by race. Family reasons were also a concern for many of the participants, especially when weight loss, changes in meal content, cost of the meals, time to cook meals, shopping for food, and time away from home became the center of discourse at home. Surprisingly to us, not everyone in the household was supportive of a change in eating behavior or in the participant’s weight loss. Again, we tried to address some of these issues with the volunteers during the consenting process. In one example, we had extended the length of the program due to a slow start in losing weight only to cancel post testing due to a tragic loss of the participant’s home to a tornado. However, attrition is a common problem with many weight loss investigations, including some of the difficulties we mention above [12,19]. A recent pulmonary rehabilitation study had a compliance rate of 85.5% (range: 72.0 – 94.4%), and only 2 out of 23 obese asthmatic patients dropped out during the 3-month intervention program [13]. It is possible that obese patients with pulmonary diseases such as asthma or COPD may be more likely to adhere to a weight loss program when compared with otherwise healthy obese adults. However, exertional dyspnea is a significant concern in otherwise healthy obese adults [20] and treatment options for dyspnea are limited to pulmonary rehabilitation or weight loss/exercise training intervention programs [21], which makes it important for researchers to consider the issues related to retention in these types of programs for otherwise healthy obese adults. We adjusted some of our program procedures to combat failure to lose weight, although failure to lose weight only accounted for 11% of attrition. Nevertheless, we believe this is an essential point to make because close monitoring of the trial, early on, seems to be extremely important. First, we added a nutritional consult with a dietician, which helped solve many of the participants’ problems with following the diet. We also added emphasis during the consenting process on how difficult it may be to lose weight and that it was necessary to attend all of the appointments with the trainers during the study. Also, we explained more about the weightlifting exercises used during the study and how difficult the exercises may feel for some participants. We increased our interaction with the participants during the weight loss program as well. We asked the individuals to meet with us at weeks 3 and 6 of the intervention. During these meetings we reviewed their progress and asked if there were any problems with following the meal plan, shopping for groceries, weighing/measuring ingredients, cooking/preparing meals, packing meals for the day, spacing meals throughout the day, water intake, changes in daily energy and sleeping patterns, training, appointment times, or any other aspects of the program in general. The investigators stayed more involved with the participants during the program to establish a greater amount of personal rapport, demonstrate a greater show of support, and to generate more enthusiasm for the participants’ successful loss of weight and healthy change of lifestyle. We wonder in hindsight whether some type of behavioral counseling might have helped the participants deal with the stress of participating in such an extensive program and with changes in eating behavior (i.e., not only for the participant but for the family). This aspect of physiological studies receives little attention but may have made this a much easier trial to complete. Lastly, the research staff also had more meetings with the training staff to get their feedback and suggestions, and to review objectives of the study and to show them preliminary results. All these changes may have helped keep unsuccessful weight loss to a minimum during the study, although we had anticipated that this would be our largest problem.

## Conclusion

In conclusion, recruiting and/or weight loss success may not present the most challenging aspects of completing a weight loss intervention study. Participant attrition during a weight loss program can occur for a wide range of other reasons including study burden, conflicts with participant employment, facility location, personal family interactions, behavioral issues, and unrelated medical issues. Many of these concerns may be combated during the consenting process and during early phases of the trial through increased investigator-participant interactions.

## Figures and Tables

**Table 1:** Original recruitment goal for the weight loss intervention.

Date	Start weight loss program	Complete weight loss	Total completed
Dec 2010	2	0	0
Jan 2011	2	0	0
Feb 2011	2	0	0
Mar 2011	2	2	2
Apr 2011	2	2	4
May 2011	2	2	6
Jun 2011	2	2	8
Jul 2011	2	2	10
Aug 2011	2	2	12
Sep 2011	2	2	14
Oct 2011	2	2	16
Nov 2011	2	2	18
Dec 2011		2	20
Jan 2012		2	22
Feb 2012		2	24

**Table 2:** Characteristics of partipants who completed, withdrew or were disqualified from the weight loss intervention.

Variables	Completed	Withdrew	Disqualified
	N = 29	N = 11	N = 13
Hisp./Cauc./AA/Asian/AI/2 or more*	5/13/8/0/2/1	3/3/4/0/1/0	1/0/11/1/0/0
Age (year)	33.0 ± 7.8	32.8 ± 6.9	32.9 ± 7.4
Height (cm)	163.2 ± 7.1	163.6 ± 4.5	162.3 ± 7.7
Weight (kg)	96.7 ± 13.9	99 ± 11.5	95.1 ± 13.5
BMI (kg/m^2^)	36.2 ± 3.5	37.2 ± 5.4	36.3 ± 6
Waist (cm)	103.5 ± 13.1	110.1 ± 8.9	109.4 ± 13
Hip (cm)	121.6 ± 9.1	122.9 ± 10.4	122.2 ± 11
Body fat (%)	47.6 ± 8.3	47.7 ± 4	46.7 ± 5.4

Hisp: Hispanic; Cauc.: Caucasian; AA: African American; AI: American Indian.

* P = 0.038
